# Is the Brood Pattern within a Honey Bee Colony a Reliable Indicator of Queen Quality?

**DOI:** 10.3390/insects10010012

**Published:** 2019-01-08

**Authors:** Kathleen V. Lee, Michael Goblirsch, Erin McDermott, David R. Tarpy, Marla Spivak

**Affiliations:** 1 Department of Entomology, University of Minnesota, 1980 Folwell Ave, Suite 219, Saint Paul, MN 55108, USA; goblirmj@umn.edu (M.G.); spiva001@umn.edu (M.S.); 2 Department of Entomology & Plant Pathology, North Carolina State University, Raleigh, NC 27695, USA; eemcderm@ncsu.edu (E.M.); drtarpy@ncsu.edu (D.R.T.)

**Keywords:** *Apis mellifera*, queen, brood pattern, queen quality, colony health, beekeeping, parasites and pathogens, pesticides

## Abstract

Failure of the queen is often identified as a leading cause of honey bee colony mortality. However, the factors that can contribute to “queen failure” are poorly defined and often misunderstood. We studied one specific sign attributed to queen failure: poor brood pattern. In 2016 and 2017, we identified pairs of colonies with “good” and “poor” brood patterns in commercial beekeeping operations and used standard metrics to assess queen and colony health. We found no queen quality measures reliably associated with poor-brood colonies. In the second year (2017), we exchanged queens between colony pairs (*n* = 21): a queen from a poor-brood colony was introduced into a good-brood colony and vice versa. We observed that brood patterns of queens originally from poor-brood colonies significantly improved after placement into a good-brood colony after 21 days, suggesting factors other than the queen contributed to brood pattern. Our study challenges the notion that brood pattern alone is sufficient to judge queen quality. Our results emphasize the challenges in determining the root source for problems related to the queen when assessing honey bee colony health.

## 1. Introduction

The queen is arguably the most important member of a honey bee colony. She is tasked with the production of daughter workers that forage for resources and care for the brood—eggs, larvae, and pupae—and sons that support genetic diversity among colonies through mating with virgin queens from other colonies. The demand by the colony placed on the queen for a sustained, high-reproductive output underscores the importance of her well-being to a colony’s success. Beekeepers are appreciative of queen health, as healthy queens ultimately lead to greater revenue generated from the sale of surplus bees, hive products, and pollination services. Beekeepers rely on various metrics associated with a queen’s reproductive output when surveying their colonies to establish the health status of their queens. They then use this information to make management decisions based on whether a queen is judged to be “good” or “failing”. However, are the signs and symptoms used to discern a good queen from a failing queen sufficient to inform management decisions? Finding an answer to this question is needed as queen health is a current issue in the beekeeping industry. Beekeepers repeatedly identify queen failure as a significant contributor to colony mortality in their responses on annual colony loss surveys, with commercial beekeepers—beekeepers that manage >500 colonies—ranking it as the first or second contributing factor [1,2].

Queens generally live one to three years. As a queen’s age increases, so does the likelihood of a supersedure event occurring—when the bees raise a new queen to replace the old queen—or the queen dying [3]. To avoid an interruption in brood production associated with aging queens, it is common practice for beekeepers to replace older queens annually. However, in recent years, beekeepers have reported queen failures after introducing young, newly mated queens into colonies. Causes for why young queens fail are not well understood. One explanation for failure observed in young queens may be a breakdown in the linkage between sperm stored in the spermatheca and successful fertilization of worker-destined eggs [4]. Approximately 10 days after emergence as an adult, queens acquire and store all of the sperm they will use throughout their lifetime from a single bout of mating with 10 to 20 drones that occurs over one or two days [5]. Inadequate sperm quantity or quality, either because of too few matings or inviable sperm in the ejaculate of the drones, could lead to elevated rates of fertilization failure, to which the colony may respond by supersedure [4]. However, sperm viability appears to be of greater concern than sperm load, as queens are often adequately mated—stored sperm counts within the queen are high [6]—but the viability of stored sperm diminishes over time [7,8]. Low viability can be an issue as the queen may have smaller brood patches [9] or can become a “drone layer”—laying only unfertilized eggs [10]. Queens inbred to related drones produce inviable worker eggs [5], although this is uncommon in commercial queen production [6,11].

Additional factors that may play a causal role in queen failure are queen infection with pathogens or exposure to pesticides. There is evidence for negative effects of pathogens on queen physiology (reviewed previously [12]). Infection with deformed wing virus has been associated with ovarian degeneration [13], and an infection of the fungal pathogen *Nosema ceranae* may lead to relatively higher vitellogenin levels [14] and upregulation of immune genes [15]. Physiological changes observed in queens exposed to different pesticides include lower queen weight [16,17] and fewer ovarioles [16]. Moreover, pesticides have been shown to affect sperm viability in queens, including in-hive chemicals used by beekeepers to control the parasitic mite *Varroa destructor* [18,19,20] and agricultural chemicals [19]. Pesticide exposure has also been linked to higher rates of queen supersedure [21,22,23] and decreased survival in combination with a *N. ceranae* infection [24].

One sign commonly attributed to failing queens is a poor brood pattern. Brood pattern refers to wax-capped cells containing pupae, also called sealed brood. A brood pattern is considered to be poor if ≥20% of the cells within an area of sealed brood are empty [25] and indicates that either the queen is not laying eggs well or the developing bees are not surviving to eclosion. In addition to queen quality measures, colony environment may influence brood pattern. Poor brood patterns have been associated with the fungal pathogen chalkbrood, sacbrood virus, and *Nosema* spp. infections of >1 million spores per bee [25]. Pesticide exposure in the comb [26] or lack of adequate nutrition may impact brood health [27], leading to decreased brood viability. In contrast, there also are heritable traits where worker bees remove diseased or *V. destructor* infested brood resulting in a worse brood pattern but a healthier colony [28,29].

The overall objective of this study was to examine if young, failing queens could be a major causal factor of poor sealed-brood patterns. In the first year of the study, we used standard metrics to assess queen and colony health in colonies with good-brood and poor-brood patterns. In the second year, we included additional measures of colony environment to begin to untangle the effects of the queen and the colony. Our specific objectives were to (1) determine if brood pattern is a reliable indicator of queen quality, (2) identify colony-level measures associated with poor brood pattern colonies, and (3) examine the change in brood patterns after queens were exchanged into a colony with the opposite brood pattern classification.

## 2. Materials and Methods

### 2.1. Colony Selection

**Figure 1:**
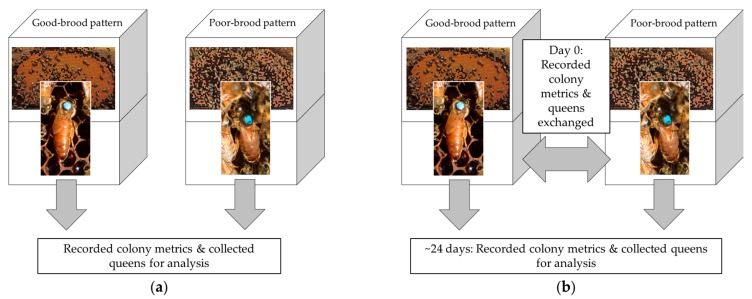
Experimental design. (**a**) In 2016, 17 poor-brood pattern and 17 good-brood pattern colonies were identified, colony metrics recorded, and queens collected and shipped live for analysis. (**b**) In 2017, 21 poor-brood pattern and good-brood pattern colony pairs were identified (42 total colonies), colony metrics recorded, and samples taken. On the same day, queens were exchanged between poor-brood pattern and good-brood pattern colony pairs. In approximately 24 days, colony metrics were again recorded, and queens collected and shipped live for analysis.

In 2016 and 2017, we identified colonies with poor sealed brood patterns and good sealed brood patterns in May and June. For each poor-brood colony, we identified a good-brood colony with the same management history within a commercial beekeeper operation based in North Dakota, Minnesota, or Texas. All colonies were headed by queens <6 months old and all queens were produced and mated in Texas or California. Data were collected from 34 colonies and queens from five operations in 2016, and 42 different colonies and queens from four operations in 2017 (Figure 1).

**Figure 2:**
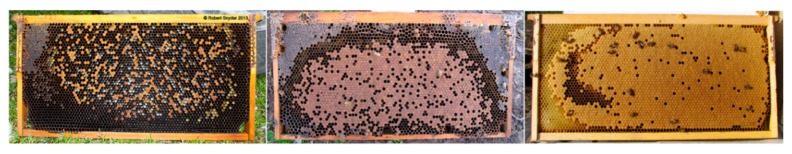
Sealed brood patterns rated a 1 (**left**), 3 (**middle**), and 5 (**right**). Photo credit: Rob Snyder.

Sealed brood patterns were rated using an ordinal scale from 1 (poor) to 5 (excellent) (Figure 2) by two field technicians with extensive experience in using the rating system (modified from a past paper [30]). A score of <3 was considered to be poor. In 2017, sealed brood pattern was also measured by quantifying the percent of sealed brood cells by placing a parallelogram large enough to occupy 100 cells over a section of sealed brood, then counting the number of empty cells—cells without sealed brood—within the parallelogram (described previously [25,31]). The number of empty cells was subtracted from 100, and the average was taken from three separate frames containing the fewest empty cells. A brood pattern with <80% sealed brood was considered to be poor [25,32]. The parallelogram method was also used to quantify the queen’s egg-laying pattern by identifying the area of comb that had the most continuous patch of eggs in each colony, counting the number of empty cells, and subtracting the number of empty cells from 100.

In 2017, we used a partial reciprocal transplant design to quantify the change in brood patterns for queens placed into different colony environments (Figure 1b). Pairs of colonies with poor-brood and good-brood patterns were identified from the same apiary or a nearby apiary with the same management history. Queens were removed and marked with a paint pen for later identification, and then placed in queen cages provisioned with food. Queens were then exchanged between colony pairs, such that a queen previously identified from a poor-brood colony was introduced into a good-brood colony and a queen from a good-brood colony was introduced into its poor-brood colony pair. Caged queens were released manually approximately 3 days after introduction into their new colony. Brood pattern measurements were recorded before the reciprocal exchange and approximately 21 days after the queen’s release to allow the queens to complete one full worker brood cycle in their new colony.

### 2.2. Queen Mating Quality and Morphometric Measurements

In 2016, queens were removed from their colonies and caged individually the same day colony metrics were recorded (Figure 1a). Cages were provisioned with food and seven worker bees from the colony where the queen was removed served as attendants. In 2017, only queens still alive after the exchange were collected. Within two days of being sampled, all queens were shipped live overnight to the North Carolina State University Queen & Disease Clinic (NCSU-QDC). At the NCSU-QDC, queens were immobilized by carbon dioxide narcosis and external morphometrics were measured: head width (mm), thorax width (mm), and wet mass (mg). The spermatheca of each queen was then extracted and the sperm within was suspended in buffer and differentially (live-dead) dyed in accordance with the procedure that accompanies the Invitrogen Live-Dead Sperm viability kit (Invitrogen L7011). Twenty microliters of the sample was then transferred to a cell counting chamber and visualized on the Nexcelom Vision^®^ System [33]. The total number of live and dead sperm were counted, and sperm viability was defined as the percent of live sperm out of the total sperm.

### 2.3. Colony Measurements

Adult bee populations were estimated by counting the number of frames in the colony that were fully covered by adult bees (described previously [31,34]). Presence of queen cells—which are indicators of swarming, supersedure, or queen loss—were noted along with any visual signs of disease or pests, including American foulbrood (*Paenibacillus larvae*), European foulbrood (*Melissococcus plutonius*), chalkbrood (*Ascosphaera apis*), sacbrood virus, hive beetles (*Aethina tumida*), wax moth (*Gallaria melonella*), *V. destructor* mites, deformed wings, and parasitic mite syndrome [35]. Entombed pollen [36] was noted if found.

From a brood frame, approximately 300 worker bees were collected into a 4oz bottle containing 70% ethanol from each colony to quantify the adult bee infestation levels of *V. destructor* and *Nosema* spp. *Varroa destructor* levels were quantified using an alcohol wash to dislodge the mites from the adult bees in the sample [37], then the mites and bees were counted and reported as mites per 100 bees. *Nosema* spp. levels were quantified by counting spores found in a composite sample of 100 bees [38]. The method of Cantwell [38] does not differentiate between *N. apis* and *N. ceranae*, the two species known to cause infection in US honey bees. If a sample was found to be positive for *Nosema* spp. infection, it was assumed to be *N. ceranae* due to findings from a recent US survey on honey bee diseases [39].

A sample of empty wax comb (>3 g) was collected into a 50 mL conical tube and stored at −80 °C before shipment on ice to the USDA-AMS lab in Gastonia, North Carolina for pesticide residue analysis. Wax samples were screened for 175 and 202 pesticides and their metabolites in 2016 and 2017, respectively (analysis methods described previously [40]) (see Supplementary Material Dataset 1: S1b. Pesticides 2016 and S1d. Pesticides 2017). Not all pesticide samples were processed due to cost. Hazard quotients (HQs) and the total number of pesticides detected were used to establish the pesticide risk in each colony. HQs were calculated by dividing the amount of the pesticide found (ppb) in the wax sample by the adult bee contact LD_50_ reported for adult honey bees (methods described previously [23,41]). The LD_50_ for each pesticide was obtained primarily by using US EPA Ecotox Database [42]. Additional resources [23,43,44] were used when the adult bee contact LD_50_ was not available through the US EPA Ecotox Database (see Table S1). Wax HQs were considered elevated if they exceeded a value of 5000 [23]. The total number of pesticide residues in the wax sample was calculated by adding the number of unique pesticides detected for each colony. The HQ and total number of pesticides detected offer an approximate measure for pesticide exposure in the colony; however, they do not account for synergistic or sublethal effects, larval toxicity, or adult oral toxicity, but both have previously been associated with queen failure [23].

### 2.4. Molecular Analysis

Total RNA was extracted from the remaining queen tissues (after dissection of the spermathecae). Queens were homogenized in individual microcentrifuge tubes with a plastic pestle in an appropriate volume of Trizol (Thermo Fisher Scientific, HQ in Waltham, MA, USA) and extraction was performed by standard phenol-chloroform protocol. Samples were then tested on the NanoDrop for quality and concentration. RNA concentration was diluted to a normalized 200 ng/uL before cDNA (Biobasic Inc. in Markham, ON, Canada) was synthesized with the BioBasic Reverse Transcriptase Mix. Reverse transcription quantitative PCR (rt-qPCR) was performed following a previously described method [45] for detection of the following pathogens: *Nosema* spp. (universal primer), trypanosome spp. (universal primer), acute bee paralysis virus (ABPV), black queen cell virus (BQCV), chronic bee paralysis virus (CBPV), deformed wing virus type A (DWV-A) and type B (DWV-B), Israeli acute paralysis virus (IAPV), and Lake Sinai virus (LSV). qPCR was performed in triplicate with Power-Up SYBRGreen Mastermix (Thermo Fisher Scientific, HQ in Waltham, MA, USA) on a 384-well QuantStudio Flex 6 (Thermo Fisher Scientific, HQ in Waltham, MA, USA) and analyzed in the associated software. Cycling conditions were adapted from the Power-Up SYBR Green protocols. The standard curve for copy number quantification was determined by running a dilution series of known plasmid standard on each plate. Results were normalized via GeNorm (reference) to the reference genes Actin, Apo28s, and GapDH, are reported as presence or absence of the pathogen. 

In 2017 before queens were exchanged between pairs of colonies, >50 adult bees were collected from a brood frame into a 50 mL conical tube from each colony. Samples were frozen immediately using dry ice or liquid nitrogen, stored at −80 °C, and then shipped on dry ice to the NCSU-QDC. Samples were analyzed by rt-qPCR for pathogens (see above), the storage protein vitellogenin (*Vg*), heat shock protein HSP70 ab-like, and the immune peptides defensin and hymenoptacin. For each colony-level sample, 5 g (approximately 50 bees) were extracted. The entire 5 g sample was homogenized in an appropriate volume of Trizol and extracted by standard phenol-chloroform extraction. The rest of the extraction was performed as above. Expression levels of the immune genes, HSP70 ab-like and *Vg* were determined via ΔΔCt analysis as compared to the reference gene Actin, not by standard curve quantitation. These genes were tested as they can indicate the health of the bees: *Vg* can influence the lifespan and decrease the oxidative stress of worker bees [46,47,48,49], relatively higher values for the immune genes suggest an upregulated immune system [50,51], and the upregulation of heat shock proteins suggests a response to stressors resulting in denatured proteins [52]. Upregulation indicates that the immune system is more active—potentially in response to a pathogen or other stressor—and is costly to the individual bee. 

### 2.5. Statistical Analysis

We used the statistical program R for all analyses [53]. All statistical assumptions were visually checked, and if violated an appropriate test was used—the nonparametric Kruskal–Wallis test or the Welch’s *t*-test for unequal variances—or the data were transformed. Summary data are reported as means ± SD unless otherwise noted. Statistical comparisons were considered significant if α < 0.05. All raw data can be found in Supplementary Material Dataset 1.

To ensure brood patterns were different between poor-brood and good-brood colonies, we compared the brood pattern scores—rating in 2016 and percent sealed in 2017—between the two groups using a Kruskal–Wallis test for the 2016 data and a Welch’s *t*-test for the 2017 data. For the 2017 data, a simple linear regression was used to examine the relationship between the two methods of measuring sealed brood patterns. 

For objectives 1 and 2, we used odds ratios (±95% confidence intervals) to compare the odds of a pathogen occurring in a poor-brood queen or colony compared to a good-brood queen or colony [54]. An odds ratio value significantly >1 indicates a positive association, and an odds ratio value significantly <1 indicates a negative association. To calculate the odds ratio in cases where no pathogen was detected, the Haldane–Anscombe correction was used [55,56]. We used *lme4* in R [53,57] to perform analyses using linear mixed effects models to compare the relationships between queen or colony measures and the binary brood pattern classification of good-brood or poor-brood. The brood pattern classification was used as a fixed effect, and beekeeper as a random factor with random slopes for the effect of the brood pattern classification. *p*-values were obtained by using likelihood ratio tests comparing the full model with the brood classification as a factor to the model without the brood pattern classification. The effect levels are reported as the estimate ± standard errors.

For objective 3, we compared the sealed brood pattern of each queen in her original colony to her sealed brood pattern approximately 21 days after being released into her new colony. We predicted that if colony environment had an effect on brood pattern, then the pattern should either improve when a queen from a poor-brood colony was placed into a good-brood colony or worsen when a queen from a good-brood colony was placed into a poor-brood colony: the change in brood pattern (after minus before the exchange) would be significantly different than zero using a *t*-test. In addition, we examined the relationship between the brood pattern before the exchange, and the change in brood pattern (after minus before the exchange) using a simple linear regression. We predicted that the queens with best or worst brood patterns before the exchange would have the largest change in brood pattern after they the queens were transferred to their reciprocal colonies. We also compared queen egg patterns before and after the exchange using a *t*-test and a simple linear regression. Data were excluded from these analyses if the queen was not found after the exchange.

## 3. Results

### 3.1. Brood Pattern Classifications

Brood patterns were significantly different between good-brood and poor-brood pattern colonies in 2016 based on the brood rating scale (*H* = 25.6, df = 1, *p* < 0.01) and 2017 based on the percent of cells sealed (t_23.93_ = 10.01, *p* < 0.01), confirming that the poor-brood and good-brood classifications were different. In 2016, the mean brood rating was for 4.0 ± 0.4 good-brood colonies (*n* = 17) and 1.9 ± 0.5 for poor-brood pattern colonies (*n* = 17). In 2017, the mean percent sealed brood was 93.0 ± 2.9% for good-brood colonies (*n* = 21) and 72.1 ± 9.1% for poor-brood colonies (*n* = 21). The brood rating scale was highly correlated to the percent brood measure in 2017 (R^2^ = 0.90, F_1,79_ = 731.2, *p* < 0.01), suggesting that the rating method sufficiently and accurately categorized brood patterns.

### 3.2. Measures Associated with Queens

Sperm number and sperm viability assessed from the queen spermathecae and queen morphometrics are summarized in Table 1. Data obtained from queens judged to be on average “high quality” from US commercial queen producers [6,11] are included for comparison. In general, the queen morphometrics, and number and viability of sperm in the spermathecae of the queens from our study were similar or higher than the previous studies. In 2017, three queens did not survive until the second sampling: one queen from a good-brood colony and two queens from poor-brood colonies. One queen from a poor-brood colony in Operation 1 in 2017 had a sperm viability of 1.0%, which was examined as a possible error as it was more than 2 standard deviations from the mean. This queen continued to lay fertilized worker bee eggs, which is contrary to what would be expected from queens with similar levels of sperm viability [9]. Due to the biological improbability of the results, the data for this queen was removed from sperm viability analyses. The percent sperm viability was not different between the two brood pattern classification groups in either 2016 (χ^2^ = 2.5, df = 1, *p* = 0.11) or 2017 (χ^2^ = 0.02, df = 1, *p* = 0.90). In 2016, queens from poor-brood colonies tended to have fewer sperm than good-brood colonies, but the difference was not significant (χ^2^ = 3.3, df = 1, *p* = 0.07). There was no difference in sperm count between brood pattern groups in 2017 (χ^2^ = 0.27, df = 1, *p* = 0.61). For both years, the average sperm count for both queen groups was over the 3 million sperm count threshold to be considered adequately mated [58] (Table 1). None of the queen mating or morphometric measures could be reliably associated with queens from poor-brood colonies.

None of the pathogens tested had significantly higher odds of being associated with queens from poor-brood pattern colonies (Table 2). The 2016 data for Operation 1 were not included in the PCR results because those samples were lost. Twenty-three percent of queens from 2016 and 78% of queens from 2017 had no pathogens detected from the panel of common honey bee pathogens used for screening. Moreover, ABPV, CBPV, trypanosomes spp., and *Nosema* spp. were not detected in any queens from 2016 or 2017. In both years, DWV-B was the most prevalent virus found in queen bees, followed by DWV-A. BQCV, LSV, and IAPV had low prevalence as they were found in only one or two queens in either 2016 or 2017.

### 3.3. Measures Associated with Colony Environment

#### 3.3.1. Adult Bee Pathogens

None of the pathogens tested had significantly higher odds of being associated with a poor-brood pattern colony (Table 2). *Varroa destructor* levels were not different between good-brood and poor-brood colonies for either year, and overall levels were low with few colonies having a mite load higher than a treatment threshold of 3 mites per 100 bees [34,59]. Worker bees from poor-brood colonies were not more likely to be over the threshold of >1 *Nosema* spp. million spores per bee as quantified by microscopy [39], nor be more likely to test positive for *Nosema* spp. as determined by PCR. In 2017, all worker bee samples tested positive for LSV and 35 samples also tested positive for *Nosema* spp. However, no 2017 queen tested positive for LSV or *Nosema* spp., suggesting that the queen was not vertically transmitting these pathogens and the workers did not transmit them to her.

#### 3.3.2. Brood Pathogens

It was not always possible to choose poor-brood colonies with no clinical signs of disease. Due to the near ubiquity of chalkbrood in 2017, we chose five good-brood and six poor-brood colonies with chalkbrood before the exchange that had ≤5 cells presenting symptoms of infection. After the exchange, 52% of good-brood and 76% of poor-brood colonies had chalkbrood symptoms. However, chalkbrood was not more likely to be found in poor-brood pattern colonies (Table 2). For comparison, only one good-brood colony had chalkbrood in 2016. No other brood diseases were found in either year.

#### 3.3.3. Worker Bee Vitellogenin, Immune Genes, and Heat Shock Protein

**Figure 3:**
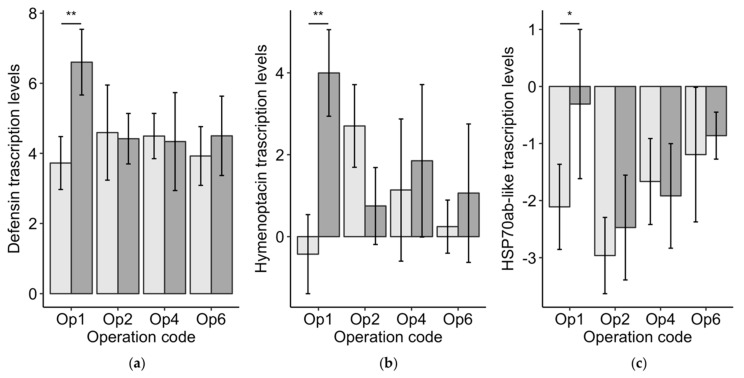
The transcription levels (means ± 95% CI)) relative to the reference gene actin for the two immune gene peptides defensin (**a**) and hymenoptacin (**b**), and the heat shock protein HSP70ab-like (**c**). The significance asterisks indicate that the only significant comparisons were between the worker bees from good-brood colonies (light grey) compared poor-brood colonies (dark grey) within Operation 1.

*Vg* levels in worker bees from poor-brood colonies were 0.90 ± 0.36 (standard error) higher than *Vg* levels in workers bees from good-brood colonies. This difference was significant (χ^2^ = 13.1, df = 1, *p* < 0.01), but may not be biologically relevant as it was under one ct cycle. We found no differences between the worker bees from good-brood and poor-brood colonies for defensin (χ^2^ = 1.3, df = 1, *p* = 0.26), hymenoptacin (χ^2^ = 0.7, df = 1, *p* = 0.39), or Hsp70ab-like (χ^2^ = 1.9, df = 1, *p* = 0.16). However, the levels of these genes were all significantly higher in Operation 1’s worker bees from poor-brood colonies (*n* = 6) compared to the worker bees from good-brood colonies (*n* = 6): defensin (*H* = 7.4, *p* < 0.01), hymenoptacin (*H* = 8.3, *p* < 0.01), and Hsp70ab-like (*H* = 5.0, *p* < 0.05) (Figure 3). *Vg* was not different between good-brood and poor-brood colonies for Operation 1 (*H* = 0.8, *p* = 0.38). No other significant differences were found for the immune genes or heat shock protein genes. These results suggest that the worker bee immune systems in Operation 1’s poor-brood colonies were upregulated.

#### 3.3.4. Colony Pesticide Levels

Twenty-eight beeswax samples were processed for pesticides in 2016 and 24 samples in 2017 (results summarized in Table S1). The pesticide data are not directly comparable between years as there were different chemicals tested each year. In 2016, there was a range of 5–16 pesticides detected per sample, and a range of 9–31 pesticides detected per sample in 2017. In 2016, the most common pesticide class found was varroacides—pesticides used to control *V. destructor*—with 44% of pesticides found belonging to this class (Figure S1). Fungicides were most common in 2017 with 45% of pesticides found belonging to that class, followed by varroacides at 24%. 

Overall HQ levels were low. Excluding Operation 5, the mean HQ in 2016 was 38 ± 71 (*n* = 22). For Operation 5, there was a high incidence of cyfluthrin (pyrethroid insecticide) in both the good-brood and poor-brood colonies resulting in higher HQs: 2093 ± 1940 (*n* = 6). One good-brood colony had an HQ >5000. In 2017, the mean HQ for good-brood colonies was 677 ± 801 (*n* = 12) and 1160 ± 894 (*n* = 12) for poor-brood colonies. All HQs in 2017 were <5000. The log transformed HQs were not significantly different between good-brood and poor-brood colonies in 2016 (χ^2^ = 0.03, df = 1, *n* = 28, *p* = 0.86) nor in 2017 (χ^2^ = 1.97, df = 1, *n* = 24, *p* = 0.16). However, the total number of pesticide residues in 2016 was significantly higher in poor-brood compared to good-brood colonies (χ^2^ = 5.00, df = 1, *n* = 24, *p* < 0.05), with poor-brood colonies having 1.9 ± 0.7 (standard errors) more pesticides detected. In 2017, there was a trend toward more pesticides in poor-brood colonies, but this result was not significant (χ^2^ = 3.8, df = 1, *n* = 28, *p* = 0.051).

### 3.4. Brood Pattern Change

**Figure 4:**
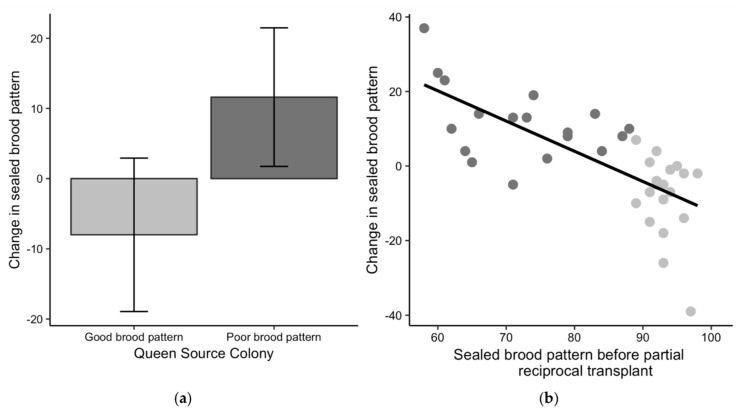
Changes in sealed brood pattern for the partial reciprocal transplant experiment in 2017. (**a**) Comparison of the change in the sealed brood pattern—a queen’s percent of sealed brood cells after the exchange minus her percent of sealed brood cells before the exchange—to the initial brood pattern classification of good (light grey) or poor (dark grey). Positive values indicate an improved brood pattern after the exchange and negative values indicate the pattern was worse after the exchange. (**b**) The potential for change in brood pattern based on the variability in the starting brood patterns.

The change in sealed brood patterns for queens from poor-brood colonies exchanged into good-brood colonies was significantly different than zero with a mean increase of 11.6 ± 9.9 more sealed cells (t_17_ = 5.0, *p* < 0.01) (Figure 4a), indicating better patterns after the exchange. The brood patterns for queens from good-brood colonies were also significantly different after the exchange into poor-brood colonies with a mean of 8.0 ± 10.9 fewer sealed cells (t_18_ = 3.2, *p* < 0.01), indicating worse patterns after the exchange. The linear regression of the starting brood pattern against the change in brood pattern was significant (R^2^ = 0.50, F_1,35_ = 36.38, *p* < 0.01), suggesting that queens with initially poor patterns tended to have improved patterns after the exchange and queens with initially better brood patterns tended to have worse patterns after the exchange (Figure 4b). This result implies that colony environment impacted the sealed brood pattern. To account for the potential effect of chalkbrood on sealed brood patterns, we removed the colonies with signs of chalkbrood after the exchange from the dataset and re-examined the relationship between the starting sealed brood pattern and the change in brood pattern. The relationship was still significant (R^2^ = 0.48, F_1,14_ = 14.99, *p* < 0.01), suggesting that the change in brood patterns was not only due to chalkbrood. 

Queens from poor-brood colonies had significantly worse egg patterns compared to queens from good-brood colonies before the exchange, with an average of 84.7 ± 16.0% sealed for poor-brood colonies compared to an average of 94.9 ± 4.7% sealed for good brood colonies (t_19.7_ = 2.6, *p* < 0.05). When the same <80% cut-off for a poor sealed brood pattern was used for the egg patterns, one queen from a good-brood colony and four queens from poor-brood colonies had “poor” egg patterns before the queen exchange. Queens from good-brood colonies transferred into poor-brood colonies had a mean egg pattern of 95.8 ± 3.4% after the exchange, and queens from poor-brood colonies transferred to good-brood colonies had a mean egg pattern of 90.8 ± 6.1%. While the difference in egg pattern was still significantly different between groups (t_26.41_ = 3.1, *p* < 0.01), only one queen, originally from a poor-brood colony, had a “poor” egg pattern of <80% after the exchange.

**Figure 5:**
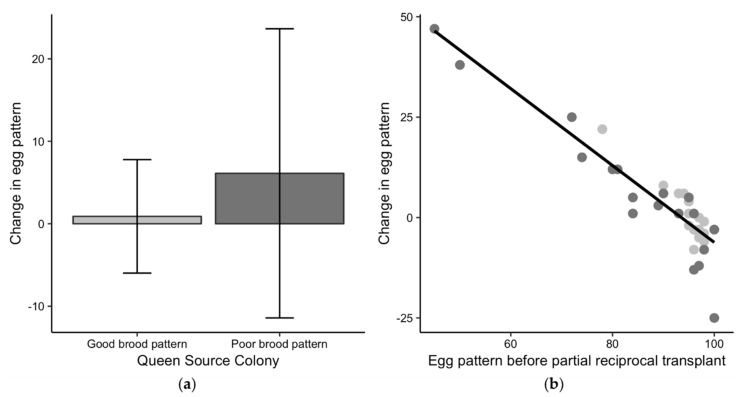
Changes in egg pattern for the partial reciprocal transplant experiment in 2017. (**a**) Comparison of the change in the egg pattern—a queen’s egg pattern after the exchange minus her egg pattern before the exchange—to the initial sealed brood pattern classification of good (light grey) or poor (dark grey). Positive values for the change in egg pattern indicate that the brood pattern improved after the exchange and negative values indicate the pattern was worse after the exchange. (**b**) The potential for change in egg pattern based on variability in the starting egg patterns.

The change in egg pattern after queens were reciprocally transferred was not different than zero for queens from either good-brood (t_18_ = 0.6, *p* = 0.58) or poor-brood colonies (t_17_ = 1.5, *p* = 0.16) (Figure 5a), suggesting that egg patterns did not change after the queen exchange based on the binary sealed brood classification. Queens from good-brood colonies had good patterns before and after they were exchanged into a potentially worse colony environment. Egg patterns for queens from poor-brood colonies did not improve on average after the exchange and the variability in egg pattern change was higher for these queens. While there was no difference in the egg pattern change when classified by the binary good or poor sealed brood classification, the queens that initially had the worst egg patterns had better patterns after being exchanged, and the queens with good egg patterns had similar or worse patterns after the exchange (R^2^ = 0.87, F_1,16_ = 115.5, *p* < 0.01) (Figure 5b). This result suggests that colony environment may have influenced the egg patterns for queens with initially the worst egg patterns as those patterns improved after the exchange. However, it is unclear why some of the good egg patterns for queens from poor-brood colonies were worse after the exchange as their egg laying potential was high.

## 4. Discussion

The results of this study suggest that a poor sealed brood pattern is not a reliable indicator of queen quality and is not necessarily a sign of queen failure. Queens from both good-brood and poor-brood colonies had sperm counts, sperm viability, body sizes, and weights that were comparable to queens considered to be of high quality in other studies [6,11]. Queens from poor-brood colonies were not more likely to have <3 million sperm in their spermathecae, which has been considered the threshold for being poorly mated [58]. There were no differences in pathogen detections between the sets of queens, including viruses, *Nosema* spp., and trypanosomes.

The partial reciprocal transplant of queens in 2017 revealed that the sealed brood patterns of queens from poor-brood colonies improved significantly after they were placed into colonies with good patterns, suggesting an influence of colony environment on the sealed brood pattern rather than solely the queens’ egg-laying capacity. None of the worker bee pathogen or immune gene measures were reliably associated with poor patterns. Levels of HQs in wax combs did not differ between brood pattern classifications. More specifically, Operation 5 reported issues with queens not being accepted by colonies in the spring of 2016; we found the highest HQs in those colonies. However, queen acceptance problems and high HQs were not found in other operations in this study. The total number of pesticides detected in wax combs was significantly higher in colonies with poorer patterns in 2016 and trended that way in 2017. Pesticide exposure may have influenced brood survivorship and thus brood pattern, but this warrants further investigation. 

In this study, we differentiated between queen and colony measures as possible causes of poor sealed brood patterns, but the queen and her colony are not mutually exclusive. Every colony phenotype is a result of both environment and genetics: how a queen’s offspring interacts with the environment, which includes nutrition, pesticides, pathogens, and beekeeper management practices. After the queen exchange in 2017, we allowed queens to lay for 21 days before removing her from the colony for sampling. It is possible that if we had left the queen in the colony and sampled after 6 weeks—when the worker bees would have been progeny of the transferred queen—that we would have been able to see if the designation of poor or good brood patterns held with the new work force. Replacing the queen could result in a better brood pattern if the colony environment remained the same and the new workers were better able to thrive in that environment.

For practical purposes, the questions important to beekeepers are action-based: under what conditions will the colony improve if the queen is replaced? Further studies on brood pattern could help elucidate the cause(s) and indicate management steps to take. A full reciprocal transplant—exchanging queens between two good-brood colonies, between two poor-brood colonies, and the same queen exchanges performed in this study—could help tease out colony vs. queen effects on brood pattern by controlling for the influence of transferring queens and the changes in environmental conditions that occur as the season progresses. Further studies could investigate colony effects on egg laying patterns by caging the queen on a frame, noting the egg pattern, then following the brood viability over time. Collecting longitudinal data on pathogens and immune genes could help determine if the brood pattern changes as these factors change. Additional measures could be included to more thoroughly judge queen quality, including the number of patrilines [60] and queen pheromone profile [61,62]. To make the study more robust, it could be done at different times of year and with different ages of queens. 

An important lesson from this study was that it was difficult to find queens with poor brood patterns without signs of brood disease. If queen failure is a leading cause of colony loss, then other symptoms besides poor brood patterns are likely to be more relevant. Beekeepers report multiple symptoms associated with younger queens failing, including stunted colony growth, relatively low brood production, irregular egg laying pattern, supersedure of apparently healthy queens, or queen death without replacement. These different symptoms may be attributed to different causes, so defining the specific symptoms and measures used to identify “failing” queens is critical to make progress in mitigating queen failures. Specifying details like queen age can make a difference in interpretations of measures like sperm viability that can decrease as queens age [7,8]. Quantifying the prevalence of different definitions of queen failure could help research target issues, and a specific definition would allow for the work to be repeatable. 

Operation 1 serves as an example of why a specific definition of queen failure matters. Operation 1 selected colonies for us to sample that matched a different definition of “queen failure”: colonies were selected based on relatively small amounts of brood—19 of approximately 800 inspected colonies—and we sampled those colonies with the worst brood patterns. In these preselected poor-brood colonies the immune systems of the worker bees were upregulated, making it appear that colony environment influenced sealed brood pattern. Because sealed brood pattern was not the primary symptom used to identify the colonies, in effect we were examining a different type of failure. The definition of “failing” used by Operation 1 may be more relevant to beekeepers, although it again may not reliably be tied to queen quality. 

## 5. Conclusions

Brood pattern alone was an insufficient proxy of queen quality. In future studies, it is important to define the specific symptoms of queen failure being studied in order to address issues in queen health.

## Figures and Tables

**Table 1:** The current study’s summary of queen quality results compared to the results from previous studies [6,11], including the number of queens tested (*n*) and the mean (±SD) values of morphometric and mating quality measures. Queens from this study are compared between brood pattern groups, with the queens from 2017 classified by their source colony status (before the exchange) of good-brood or poor-brood.

Paper (Year)	Brood Pattern	*n*	Sperm Count, Millions (Range)	Poorly Mated ^1^	Sperm Viability (%)	Weight (mg)	Thorax Width (mm)	Head Width (mm)
This study (2016)	Good-brood	17	6.74 ± 1.95 (2.55–9.37)	6%	83.7 ± 6.3	223.9 ± 17.1	4.89 ± 0.15	3.79 ± 0.14
	Poor-brood	17	5.07 ± 2.51 (0.52–8.09)	24%	78.3 ± 11.2	216.5 ± 23.0	4.85 ± 0.18	3.8 ± 0.12
This study (2017)	Good-brood	19	5.69 ± 1.82 (1.39–8.4)	11%	78.0 ± 6.4	223.6 ± 27.9	4.93 ± 0.19	3.83 ± 0.10
	Poor-brood	18	5.88 ± 1.57 (2.8–8.1)	6%	78.3 ± 10.9 ^2^	231.8 ± 23.0	4.94 ± 0.19	3.83 ± 0.09
Delaney et al. (2011)	NA	114	3.99 ± 1.50 (0.2–9.0)	18.6%	NA	184.8 ± 21.7	4.35 ± 0.19	3.62 ± 0.12
Tarpy et al. (2012)	NA	61	4.37 ± 1.45	13.6%, & 1 virgin queen	83.7 ± 3.3	218.7 ± 20.7	4.34 ± 0.23	3.45 ± 0.23

^1^ Percent of queens tested that had <3 million sperm in their spermatheca [58]. ^2^ Queen with 1% sperm viability removed from summary.

**Table 2:** Summary of the odds ratios (95% CI range) and the percent of positive pathogen detections using PCR for worker bee samples (5 g composite sample) in 2017 and queen samples in 2016 and 2017 from colonies with good-brood or poor-brood patterns. Only pathogens with positive detections are included; chronic bee paralysis virus was not found in any samples. Also included are the comparisons between poor-brood and good-brood colonies with symptoms of the brood disease chalkbrood, and worker bee samples with *Varroa destructor* mite levels >3 mites per 100 bees, and *Nosema* spp. levels >1 million spores per bee as determined by microscopy. No pathogen had significantly higher odds of being in a poor-brood pattern colony or queen.

		% of Samples with Positive Detections		
Sample Type (Year)	Factor	Good-Brood	Poor-Brood	Odds Ratio (95% CI)
Queens (2016)	No pathogens detected	33	13	0.31 (0.05–1.93)
	Black Queen Cell Virus	7	0	0.31 (0.01–8.29)
	Deformed Wing Virus type A	40	60	2.25 (0.52–9.70)
	Deformed Wing Virus type B	53	73	2.41 (0.52–11.1)
	Lake Sinai Virus	13	0	0.17 (0.01–3.96)
Queens (2017) ^1^	No pathogens detected	79	78	0.93 (0.2–4.47)
	Deformed Wing Virus type A	5	11	2.25 (0.19–27.22)
	Deformed Wing Virus type B	16	17	1.07 (0.19–6.13)
	Israeli Acute Paralysis Virus	5	0	0.33 (0.01–8.73)
Worker bees (2016)	>3 *Varroa* mites per bee	6	6	1.00 (0.06–17.41)
	>1 million *Nosema* spores per bee, by microscopy	12	18	1.61 (0.23–11.09)
Worker bees (2017) ^2^	>3 *Varroa* mites per bee	0	5	3.15 (0.12–81.74)
	>1 million *Nosema* spores per bee, by microscopy	33	48	1.82 (0.52–6.33)
	Acute Bee Paralysis Virus	10	5	0.48 (0.04–5.68)
	Black Queen Cell Virus	38	19	0.38 (0.09–1.55)
	Deformed Wing Virus type A	5	14	3.33 (0.32–34.99)
	Deformed Wing Virus type B	24	43	2.40 (0.64–9.03)
	Israeli Acute Paralysis Virus	19	5	0.21 (0.02–2.09)
	Lake Sinai Virus	100	100	1.00 (0.02–52.74)
	Trypanosomes	10	19	2.24 (0.36–13.78)
	*Nosema* spp., by PCR	90	86	0.63 (0.09–4.23)
Brood disease (2016)	Chalkbrood	6	0	0.31 (0.01–8.27)
Brood disease (2017)	Chalkbrood ^3^	52	76	2.91 (0.78–10.89)

^1^ Queens in 2017 were sampled after the queen exchange but classified by their source colony status (before the exchange) of good-brood or poor-brood. ^2^ Sampled before the queen exchange. ^3^ Accounts for chalkbrood found before and/or after queen exchange.

## Supplementary Materials

The following are available online at http://www.mdpi.com/2075-4450/10/1/12/s1, Table S1: Pesticide summary for good-brood and poor-brood colonies in 2016 and 2017, Figure S1: The relative percent of pesticide classes found in beeswax samples from 2016 and 2017, and Supplementary Materials Dataset 1: S1a. Data 2016, S1b. Pesticides 2016, S1c. Data 2017, S1d. Pesticides 2017.
