# Supplementary material for: Is the Brood Pattern within a Honey Bee Colony a Reliable Indicator of Queen Quality?
Source: Insects. 2019 Jan 8;10(1):12. doi: 10.3390/insects10010012 (PMC6359415; doi:10.3390/insects10010012)
Supplement: Supplementary file 1 [file insects-10-00012-s001.zip › Supplementary material/Table S1.pdf]

Lee, K., Goblirsch, M., McDermott, E., Tarpy, D.R., Spivak, M. Is the brood pattern within a honey bee colony a reliable indicator of queen quality?

Table S1. Summary of each pesticide found in wax samples from colonies with good sealed brood patterns (2016: n = 14; 2017: n = 12) and poor sealed brood patterns (2016: n = 14; 2017: n = 12), including the mean ppb, mean hazard quotient (HQ), limit of detection (LOD) for 2016 and 2017, pesticide class, adult bee contact LD_{50}, and the percent of positive detections. The adult bee contact LD_{50} was determined using the US EPA Ecotox Database [42] unless otherwise noted.

				2016						2017					
				Good-brood colony			Poor-brood colony			Good-brood colony			Poor-brood colony		
**Pesticide**	**LOD (ppb), 2016/2017 (NT = not tested)**	**Class[1]**	**LD_{50}**	**Mean ppb**	**Mean HQ**	**% pos. detections**	**Mean ppb**	**Mean HQ**	**% pos. detections**	**Mean ppb**	**Mean HQ**	**% pos. detections**	**Mean ppb**	**Mean HQ**	**% pos. detections**
2,4 Dimethylphenyl formamide (DMPF)	5/1.5	VARRO	75[3]	60.4	0.8	100	61.1	0.8	100	326.1	4.0	100	452.7	6.0	100
Acetochlor	15/100	HERB	1715							16.7	0.0	17	16.7	0.0	17
Atrazine	4/4	HERB	97	0.2	0.0	6	0.0	0.0	0			0			0
Azoxystrobin	5/1	FUNG	200							2.9	0.0	50	6.1	0.0	75
Bentazon	NT/30	HERB	100							0.0	0.0	0	2.5	0.0	8
Boscalid	10/5	FUNG	200	4.2	0.0	35	5.9	0.0	41	10.8	0.1	92	26.0	0.1	83
Buprofezin	60/2	IGR	200							1.3	0.0	33	31.9	0.2	67
Captan	50/250	FUNG	215	0.0	0.0	0	8.8	0.0	6			0			0
Carbaryl	2/5	INSECT	1.1	2.4	2.1	18	0.3	0.3	12	0.0	0.0	0	11.0	10.0	17
Carbendazim MBC	5/2	FUNG	50	1.0	0.0	18	6.6	0.1	41	18.4	0.3	50	6.5	0.1	67
Chlorothalonil	100/250	FUNG	181.3							41.7	0.2	17	62.5	0.3	25
Chlorpropham CIPC	10/15	HERB	36.6							6.1	0.2	25	100.3	2.7	25
Chlorpyrifos	5/5	INSECT	0.01							5.9	546.2	67	9.2	916.7	83
Chlorthal-dimethyl DCPA	NT/2	HERB	100[4]							0.0	0.0	0	0.6	0.0	17
Coumaphos	3/4	VARRO	20[5]	42.2	2.1	94	48.9	2.4	88	226.1	10.4	83	617.8	30.9	100
Coumaphos oxon	2/0.5	VARRO	20[5]	2.4	0.1	71	4.6	0.2	71	10.6	0.5	75	20.6	1.0	83
Cyfluthrin total	10/25	INSECT	0.037	19.4	523.1	24	8.9	240.1	18						
Cyhalothrin total	5/100	INSECT	0.022	1.2	53.5	6	0.7	32.1	6			0			0
Cyprodinil	10/2	FUNG	784	10.5	0.0	29	87.1	0.1	71	30.5	0.0	83	42.5	0.1	92
DDE, p,p'	5/2	INSECT	6.4[3]							0.3	0.0	17	0.3	0.1	17
Difenoconazole	10/4	FUNG	101	1.2	0.0	12	1.4	0.0	6						
Diflubenzuron	5/2	IGR	114	7.1	0.1	35	17.4	0.2	65	6.3	0.1	42	13.0	0.1	75

Diuron	NT/1	HERB	145							0.1	0.0	8	0.4	0.0	25
Endosulfan II	10/10	INSECT	7.1							0.4	0.1	8	0.0	0.0	0
Ethion	15/25	INSECT	11[5]							0.0	0.0	0	2.1	0.2	8
Fenamidone	30/1	FUNG	47.1							0.2	0.0	17	0.2	0.0	17
Fenbuconazole	15/3	FUNG	292	1.6	0.0	12	1.8	0.0	12	0.9	0.0	25	11.3	0.0	50
Fenpyroximate	4/3	VARRO	40	64.9	1.6	82	140.6	3.5	65	215.7	5.0	83	200.1	5.0	67
Fluopyram	5/1	FUNG	100	0.0	0.0	0	1.5	0.0	12	4.0	0.0	58	2.2	0.0	83
Fluvalinate	5/25	VARRO	8.1	132.4	16.3	71	189.5	23.4	65	117.5	13.4	100	446.8	55.2	100
Fluxapyroxad	5/2	FUNG	370.4							5.8	0.0	67	4.8	0.0	83
Hexythiazox	15/2	MITI	200							0.5	0.0	25	0.0	0.0	0
Imidacloprid	6/5	NEONIC	0.036	0.4	9.8	6	0.0	0.0	0						
Indoxacarb	30/7	INSECT	0.18	0.0	0.0	0	2.8	15.4	6			0			0
Iprodione	15/100	FUNG	200							442.9	2.0	83	571.3	2.9	100
Malathion	10/25	INSECT	0.64	0.9	1.4	6	0.0	0.0	0						
Mandipropamide	NT/2	FUNG	200							0.0	0.0	0	0.2	0.0	8
Metalaxyl	5/1	FUNG	100	0.0	0.0	0	0.3	0.0	6						
Metconazole	10/5	FUNG	100[4]	0.6	0.0	6	8.4	0.1	18	2.1	0.0	25	1.8	0.0	33
Methoxyfenozide	5/1	IGR	100	3.7	0.0	24	14.6	0.1	71	8.9	0.1	92	20.3	0.2	100
Metolachlor	5/25	HERB	110							6.3	0.1	25	8.3	0.1	33
MGK 264[2]	25/7	INS SYN								1.1	0.0	8	4.3	0.0	17
Myclobutanil	15/7	FUNG	33.9[4]							2.3	0.1	8	0.0	0.0	0
Novaluron	NT/5	IGR	100							0.0	0.0	0	1.0	0.0	17
Oxyfluorfen	5/100	HERB	100	1.4	0.0	18	1.1	0.0	12	0.0	0.0	0	10.8	0.1	8
Pendimethalin	15/50	HERB	100[4]							31.4	0.3	67	43.1	0.4	92
Penthiopyrad	NT/1	FUNG	500							1.0	0.0	17	7.3	0.0	67
Piperonyl butoxide	11/12	INS SYN	11	175.3	15.9	47	35.3	3.2	24	58.1	4.9	100	80.2	7.3	100
Prometon	NT/1	HERB	36							0.1	0.0	8	0.0	0.0	0
Propargite	15/2	MITI	15							1.4	0.1	17	0.0	0.0	0
Propiconazole	15/2	FUNG	25	2.6	0.1	18	22.9	0.9	41	29.1	1.1	92	13.3	0.5	92
Pyraclostrobin	5/2	FUNG	100	10.6	0.1	82	17.0	0.2	88	18.2	0.2	83	36.5	0.4	92
Pyridaben	5/2	INSECT	0.024							0.0	0.0	0	0.1	3.5	8

Pyrimethanil	100/5	FUNG	100	1.5	0.0	6	6.2	0.1	18	5.8	0.1	42	12.0	0.1	42
Pyriproxyfen	5/1	IGR	74	1.2	0.0	24	0.6	0.0	12	0.2	0.0	8	2.6	0.0	33
Spinetoram	NT/15	INSECT	0.024[4]							0.0	0.0	0	1.3	52.1	8
Spinosad	15/7	INSECT	0.047							1.8	34.4	25	2.9	62.1	42
Tebuconazole	15/5	FUNG	200[4]	2.6	0.0	18	7.6	0.0	47	3.8	0.0	42	8.7	0.0	50
Tetraconazole	15/2	FUNG	63[4]	1.8	0.0	12	0.9	0.0	6						
Thymol	50/2	VARRO	1273[4]	284.1	0.2	29	532.4	0.4	24	1582.0	1.1	100	1574.4	1.2	100
Trifloxystrobin	10/1	FUNG	200	0.0	0.0	0	2.2	0.0	6	6.0	0.0	58	7.1	0.0	83
Triflumizole	40/1	FUNG	160							0.2	0.0	17	0.1	0.0	8

[1] HERB = herbicide; FUNG = funigicide, IGR = Insect Growth Regulator; INSECT = insecticide; INS SYN = insecticide synergist; MITI = miticide; NEONIC = neonicotinoid insecticide; VARRO: varroacides applied by beekeepers for control of *V. destructor*.

[2] Not included in HQ calculations as it has no reported honey bee contact LD_{50}.

[3] LD_{50} determined using [23].

[4] LD_{50} determined using [43].

[5] LD_{50} determined using [44].
