# Supplementary material for: Is the Brood Pattern within a Honey Bee Colony a Reliable Indicator of Queen Quality?
Source: Insects. 2019 Jan 8;10(1):12. doi: 10.3390/insects10010012 (PMC6359415; doi:10.3390/insects10010012)
Supplement: Supplementary file 1 [file insects-10-00012-s001.zip › Supplementary material/Figure S1.pdf]

Lee, K., Goblirsch, M., McDermott, E., Tarpy, D.R., Spivak, M. Is the brood pattern within a honey bee colony a reliable indicator of queen quality?

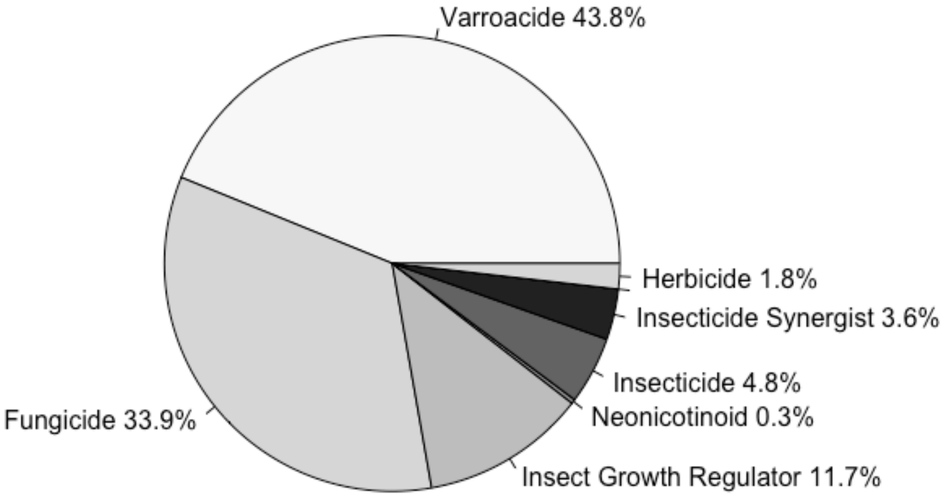

(**a**)

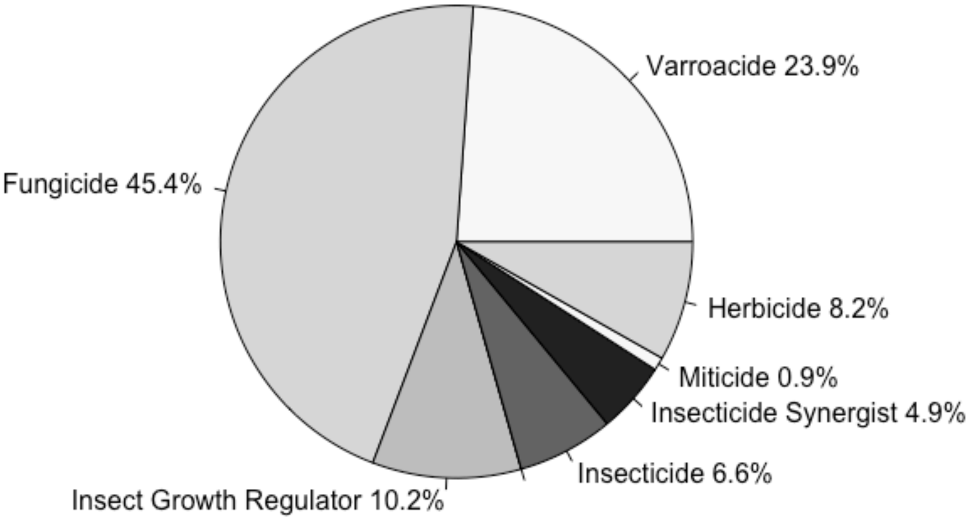

(**b**)

Figure S1. The relative percent of pesticide classes found in beeswax samples from (a) 2016 and (b) 2017.
